# Cookery

**Published:** 1867-12

**Authors:** 


### HALL'S JOURNAL OF HEALTH.

Our Legitimate Scope is almost boundless; for whatever begets pleasurable and harmless feelings, promotes Health; and whatever induces disagreeable sensations, engenders Disease.

WE AIM TO SHOW HOW DISEASE MAY BE AVOIDED, AND THAT IT IS BEST, WHEN SICKNESS COMES, TO TAKE NO MEDICINE WITHOUT CONSULTING A PHYSICIAN.

VOL. XIV.] DECEMBER, 1867. [No. 12.

### COOKERY.

The first, the indispensable requisites of healthful cooking are that the materials should be fresh, perfect, and ripe, and that they should be properly cooked. It may be safe to say that half of the food prepared for American tables is ruined, for all purposes of healthful nutrition, in the cooking, and destroys rather than builds up; weakens, instead of imparting vigor, and engenders wasting disease, rather than promotes good health. The higher classes of society, the best informed, appreciate these truths and seek to secure all their advantages by a more strict personal attention to the larder and the kitchen than those below them in the social scale; hence Professor Blot's observation that wherever he has gone to give practical scientific lectures on cooking, nine-tenths of his patrons have been among the elite of society; the wealthiest and the best informed, and this, too, when the price of admission has been so low as not to make it burdensome to the shortest, shallowest purse; and yet it is more important to the poor to regard cooking as a science than to the rich, because it is a true economy as well as a power to keep off expensive disease; for the poor, sickness is of double expense, time is lost and their wages stop and the little store they may have laid up is diminished by the purchase of medicine and medical advice. Many will learn with great satisfaction that the eminent able and gentlemanly Professor will issue shortly, from the press of the Appletons, if we remember rightly, a volume devoted to the scientific treatment of what is called good cookery. Much has been said in derision of French cooking, but by persons who are altogether ignorant of the subject theoretically and practically; for true French cookery comprises four things:

First, to make the poorest food palateable;

Second, to get the most nourishment out of the least quantity.

Third, to "utilize" every scrap, so that nothing is lost;

Fourth, to avoid the use of fats, fries and spices.

As proof of this the following remarks of the courtly professor, as made in a lecture at the Cooper Institute, are here given. In speaking of the advantages of

